# Pediatric Nuclear Medicine Examinations and Subsequent Risk of Neoplasm: A Nationwide Population-Based Cohort Study

**DOI:** 10.3389/fmed.2021.764849

**Published:** 2021-12-20

**Authors:** Mei-Kang Yuan, Shih-Chieh Chang, Mei-Chun Yuan, Ning-Ping Foo, Shan-Ho Chan, Shyh-Yau Wang, Cheng-Li Lin, Chung-Y. Hsu, Chia-Hung Kao

**Affiliations:** ^1^Department of Radiology, An Nan Hospital, China Medical University, Tainan, Taiwan; ^2^School of Medicine, College of Medicine, China Medical University, Taichung, Taiwan; ^3^Department of Medical Imaging and Radiology, Shu-Zen Junior College of Medicine and Management, Kaohsiung, Taiwan; ^4^Division of Chest Medicine, Department of Internal Medicine, National Yang Ming Chiao Tung University Hospital, Yilan, Taiwan; ^5^Department of Critical Care Medicine, National Yang Ming Chiao Tung University Hospital, Yilan, Taiwan; ^6^Faculty of Medicine, College of Medicine, National Yang Ming Chiao Tung University, Taipei, Taiwan; ^7^Department of Information Management, Meiho University, Pingtung, Taiwan; ^8^Department of Emergency Medicine, An Nan Hospital, China Medical University, Tainan, Taiwan; ^9^Graduate Institute of Medical Sciences, Chang Jung Christian University, Tainan, Taiwan; ^10^Management Office for Health Data, China Medical University Hospital, Taichung, Taiwan; ^11^Graduate Institute of Biomedical Sciences, College of Medicine, China Medical University, Taichung, Taiwan; ^12^Center of Augmented Intelligence in Healthcare, China Medical University Hospital, Taichung, Taiwan; ^13^Department of Nuclear Medicine and PET Center, China Medical University Hospital, Taichung, Taiwan; ^14^Department of Bioinformatics and Medical Engineering, Asia University, Taichung, Taiwan

**Keywords:** nuclear medicine (NM), pediatric neoplasms, radiation protection, malignancy, National Health Insurance Research Database (NHIRD)

## Abstract

**Objective:** To evaluate the association between radiation exposure from repeated nuclear medicine (NM) examinations and the subsequent risk of neoplasm in pediatric patients.

**Methods:** From 2000 to 2017, participants under 18 years of age who underwent NM scanning were identified using the Health and Welfare Data Science Center (HWDC) dataset, which was extracted from the Taiwan National Health Insurance Research Database (NHIRD). Both the exposed cohort and unexposed subjects were followed up with until the presence of any malignancy arose, including malignant brain, lymphoid and hematopoietic tumors and benign brain or other central nervous tumors.

**Results:** There were 35,292 patients in the exposed cohort and 141,152 matched subjects in the non-exposed group. The exposed cohort had an overall higher IR (IR: incidence rate, per 100,000 person-years) of any malignancy and benign central nervous tumor than the non-exposed group [IR, 16.9 vs. 1.54; adjusted hazard ratio (HR), 10.9; 95% CI, 6.53–18.2]. Further stratifying the number of NM examinations into 1-2, 3-4, and 5 or more times revealed that the IR of pediatric neoplasms increased gradually with the increased frequency of NM examinations (IR, 11.5; adjusted HR, 7.5; 95% CI, 4.29–13.1; IR, 25.8; adjusted HR, 15.9; 95% CI, 7.00–36.1; IR, 93.8; adjusted HR, 56.4; 95% CI, 28.8–110.3).

**Conclusion:** NM examination is significantly associated with a higher risk of pediatric neoplasms, according to our population-based data. Thorough radiation protection and dose reduction in pediatric NM procedures should be an issue of concern.

## Introduction

Radiation hazards to patients and medical workers exposed to radiological examinations have raised worldwide concerns in recent decades (1–3). The ICRP 2015 Proceedings indicate that 32.7 million global diagnostic nuclear medicine (NM) examinations are performed annually, with an increase of 0.2 million examinations per year since 1991 (4). According to the National Council on Radiation Protection and Measurements (NCRP) 160 report, NM procedures have increased from 6.3 million in 1984 to 18 million in 2006, with ~1% of these procedures performed on children in the United States (5). NM imaging provides essential physiological and pathological information in oncology, urology, and orthopedics, and such imaging is particularly valuable in children and infants, in whom a rapid and accurate diagnosis is crucial for developmental health and reducing disease progression. Children have a higher risk for adverse effects from radiation exposure than adults, and the subsequent lifelong estimated cancer risks due to repeated radiological examination should not be ignored (6, 7). NM procedures are traditionally thought to be safe and non-invasive without serious complications. For example, ^99m^Tc-DMSA renal scintigraphy can be used acutely to confirm the presence of acute pyelonephritis or, 4–6 months later, the sequelae of renal cortical scarring in pediatric patients with recurrent urinary tract infections. The estimated effective dose administered for a common ^99m^Tc-DMSA scan is low, varying between 0.6 and 0.8 millisieverts (mSv). Although the majority of NM exams performed on children require a low effective dose of <1 mSv, radiation exposure and cancer risk from repeated scans in the acute stage and disease follow-up should never be overlooked (8). Some NM examinations, especially in oncology and orthopedics, can result in an intermediate radiation dose, between 3 and 6 mSv, which is close to the radiation dose received from one modern CT scan (9, 10). Several studies have revealed evidence of higher cancer risk after repeated CT scan exposure in pediatric patients (11–15). It is conceivable that the cancer risk associated with NM procedures in the pediatric population is a topic that warrants further assessment. Our encrypted identification and medical records of participants were representatively extracted from the NHIRD, which has the advantage of being one of the largest national databases in the world. This population-based study was designed to investigate the possible association between pediatric neoplasms and diagnostic NM procedures.

## Materials and Methods

### Data Source

This study used data from the Health and Welfare Data Science Center (HWDC), which contains over 99% of 23 million Taiwanese residents' national electronic medical records. The International Classification of Diseases, Clinical Modification (ICD-9-CM) and International Classification of Diseases, Tenth Revision, Clinical Modification (ICD-10-CM) were used as diagnostic tools for diseases. This study was approved by the Central Regional Research Ethics Committee, China Medical University, Taichung, Taiwan (CMUH109–109-REC2–031).

### Study Population

**Figure 1:**
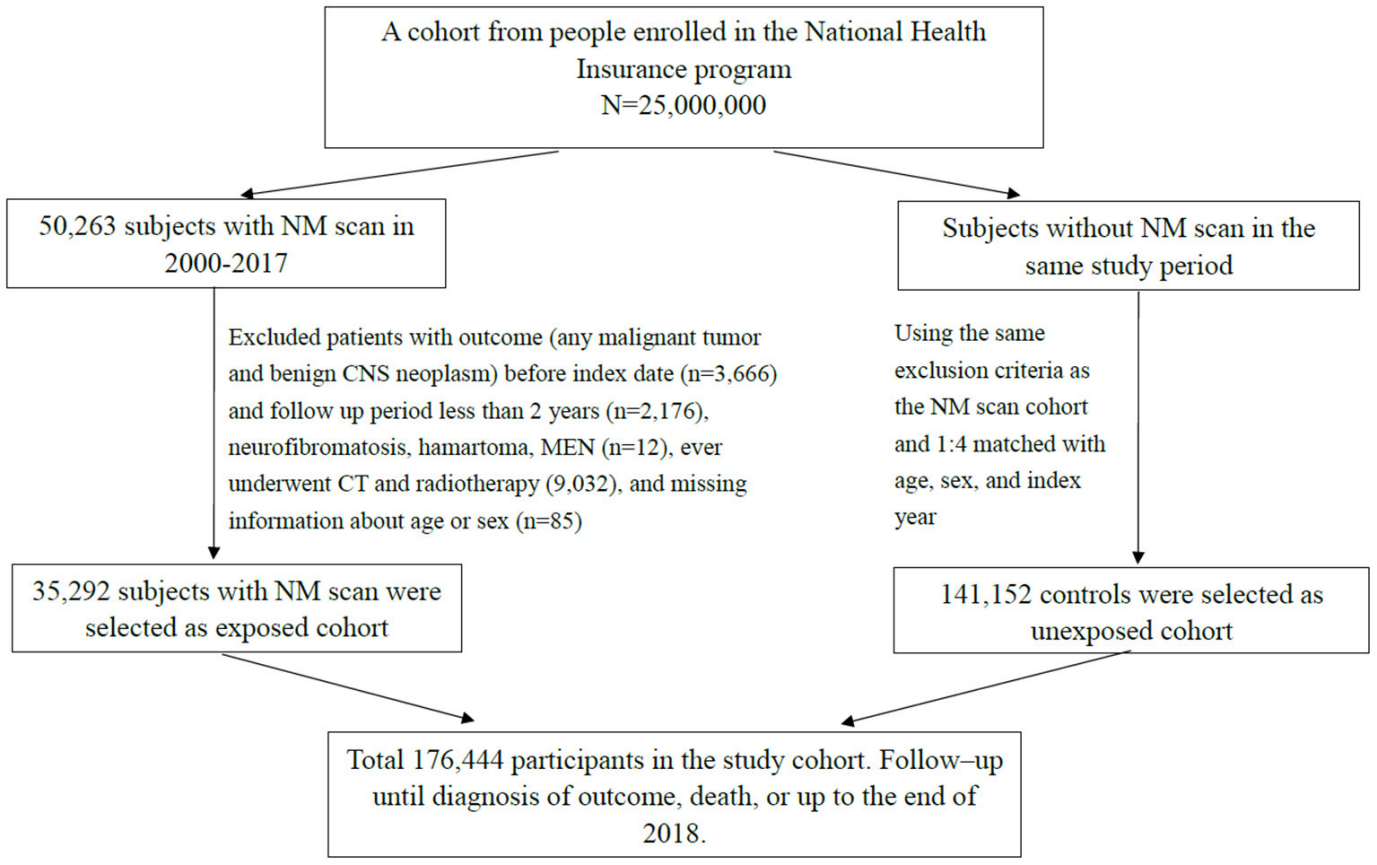
Flowchart for selecting study participants. NM, nuclear medicine; CNS, central nervous system; MEN, multiple endocrine neoplasia.

The study period of this retrospective population-based cohort study was between 2000 and 2017. We selected participants under 18 years of age who underwent nuclear medicine (NM) scans (Taiwan National Health Insurance payment code 26001B~26070B). Positron emission tomography (PET) and I^131−^related therapy were not included due to cancer selection bias. For each NM scan patient, we selected four patients who never underwent NM scans as unexposed comparison group based on sex, age, and index year. The exclusion criteria for both the NM scan group and the unexposed group were as follows: patients who ever underwent high radiation dose examination or therapy, such as CT scan and radiation therapy, and those with disorders that may have increased cancer risk, such as multiple endocrine neoplasia (ICD-9-CM code 258.01–258.03; ICD-10-CM code E31.20-E31.23); neurofibromatosis; phakomatosis; and tuberous sclerosis (ICD-9-CM code 237.70–72; 759.5, 759.6; ICD-10-CM code Q85.0–85.9). In addition, we excluded patients if they had a prior history of cancer before the index date and cancer development within the first 2 years of follow-up (lag period of 2 years for cancer diagnosis). The date for the first NM examination was defined as the index date and the start of follow-up set at 2 years after the index date. All participants were followed from cohort entry until the presence of the primary outcome, death, censoring for loss or the end of the study period (December 31, 2018), whichever came first (Figure 1).

### Outcomes

The major categories of leukemias, lymphomas and malignant brain tumors represent close to 70% of all pediatric cancers (16).

A previous study revealed that repeated head CT scans might increase the risk of benign pediatric brain tumors (12). Therefore, our primary outcome measure included any malignant tumor (ICD-9-CM code 140.0–208.92; ICD-10-CM code C00.1-C80.1), malignant lymphoid and hematopoietic tissue (ICD-9-CM code 200.00–208.92; ICD-10-CM code C81.00-C96.9), malignant brain and other central nervous tumor (ICD-9-CM code 191.0, 192, 194.2, 194.3, 194.4; ICD-10-CM code C71.0-C72.9), and benign brain and other central nervous neoplasm (ICD-9-CM code 225.0–225.9, 227.3, 227.4; ICD-10-CM code D32.0-D33.9).

### Statistical Analysis

We computed the categorical variables as numbers and percentages and the continuous variables as the mean and standard deviation (SD). For the difference in the categorical and continuous demographic variables between the NM scan group and unexposed comparison group, statistical significance was determined with the chi-square test and Student's *t*-test. To address the concern of constant proportionality, we examined the proportional hazard model assumption using a test of scaled Schoenfeld residuals. Results showed that there was no significant relationship between Schoenfeld residuals for radiation exposure and follow-up time (*p*-value = 0.71) in the model evaluating the neoplasm risk. Univariable and multivariable Cox proportional hazards models were used to estimate the risk of pediatric neoplasms in children with and without NM scans. With adjustment for sex, age, and urbanization in multivariable analysis, we obtained the adjusted hazard ratio. The Kaplan–Meier method was applied to obtain the cumulative incidence curve. Statistical analysis was performed using SAS software, version 9.4, and we generated survival curves in R software. The significance criterion was a two-sided *p*-value < 0.05.

## Results

### Characteristics of Study Population

The comparisons of demographic characteristics in participants with and without NM scans of the study are listed in Table 1. From 2000 to 2017, the cohort consisted of 35,292 patients with NM scans and 141,152 patients without NM scans. After matching, the distribution of sex, age and urbanization was similar in the NM scan-exposed group and the unexposed comparison group. The average age in both groups was ~6 years old. There were more female patients (50.4%) than male patients (49.6%) in both groups. The average follow-up time was ~9 years.

**Table 1:** Demographic characteristics and incidence rate in cohorts with and without NM examination exposure.

	**Unexposed**	**Exposure**	***P-*value**
**Variables**	**(*N* = 141,152)**	**(*N* = 35,292)**	
	***n* (%)**	***n* (%)**	
Sex			0.99
Female	71,128 (50.4)	17,784 (50.4)	
Male	70,024 (49.6)	17,508 (49.6)	
Age stratified			0.99
<12	110,689 (78.4)	27,675 (78.4)	
≥12	30,463 (21.6)	7,617 (21.6)	
Age, mean ± SD *^a^*	6.29 ± 5.23	6.16 ± 5.35	<0.001
Age, median (IQR)	4 (2–10)	4 (1–10)	
Urbanization^†^			0.02
1 (Very high)	78,165 (55.4)	19,490 (55.2)	
2	50,805 (36.0)	12,756 (36.1)	
3	9,334 (6.61)	2,415 (6.84)	
4 (Low)	2,848 (2.02)	631 (1.79)	
Follow-up year, mean ± SD^a^	9.21 ± 3.65	9.22 ± 3.63	0.95
Follow-up year, median (IQR)	9.71 (6.12–12.4)	9.75 (6.14–12.4)	

*NM, nuclear medicine*;

a *t-test, SD, standard deviation; IQR, interquartile range*.

† *The urbanization level was categorized by the population density of the residential area into 4 levels: level 1 as the most urbanized region and level 4 as the least urbanized region*.

### Higher Cancer Incidence Rates in the NM Scan Exposure Cohort

As shown in Table 2, the overall incidence rates (IR: incidence rate, per 100,000 person-years) of any malignant tumor and benign central nervous neoplasm in the NM-exposed cohort were significantly higher than those in the non-exposed group [IR, 16.9 vs. 1.54; adjusted hazard ratio (HR), 10.9; 95% CI, 6.53–18.2]. The incidence rate of malignant lymphoid and hematopoietic cancer in the exposed cohort was significantly higher than that in the non-exposed group (IR, 6.46 vs. 0.92; adjusted HR, 6.77; 95% CI, 3.33–13.8). The incidence rate of malignant brain and other central nervous tumors in the exposed cohort was significantly higher than that in the non-exposed group (IR, 2.77 vs. 0.38; adjusted HR, 6.78; 95% CI, 2.27–20.3). The incidence rate of benign brain and other central nervous tumors in the exposed cohort was significantly higher than that in the non-exposed group (IR, 1.54 vs. 0.08; adjusted HR, 25.0; 95% CI, 3.01–207.7).

**Table 2:** Comparison of incidence rate of radiosensitive tumor and hazard ratio between cohorts with or without NM procedures.

**Variable**	**Without NM scan**		**With NM scan**		**Compared to without NM scan**	
	**Event**	**IR**	**Event**	**IR**	**Crude HR (95% CI)**	**Adjusted HR (95% CI)**
Any malignant tumor and benign central nervous neoplasm	20	1.54	55	16.9	11.0 (6.58–18.3)^***^	10.9 (6.53–18.2)^***^
Any malignant tumor	19	1.46	50	15.4	10.5 (6.19–17.8)^***^	10.4 (6.10–17.6)^***^
Malignant lymphoid and hematopoietic cancer	12	0.92	21	6.46	6.99 (3.44–14.2)^***^	6.77 (3.33–13.8)^***^
Malignant brain and other central nervous neoplasm	5	0.38	9	2.77	7.19 (2.41–21.5)^***^	6.78 (2.27–20.3)^***^
Benign brain and other central nervous neoplasm	1	0.08	5	1.54	23.9 (2.88–198.6)^**^	25.0 (3.01–207.7)^**^

*NM, nuclear medicine; IR, incidence rate, per 100,000 person-years; HR, hazard ratio; Adjusted HR, multivariable analysis including sex, age, urbanization*.

** *p < 0.01*.

*** *p < 0.001*.

### Trend in Increased NM Frequency Associated With Higher Cancer Risk

**Figure 2:**
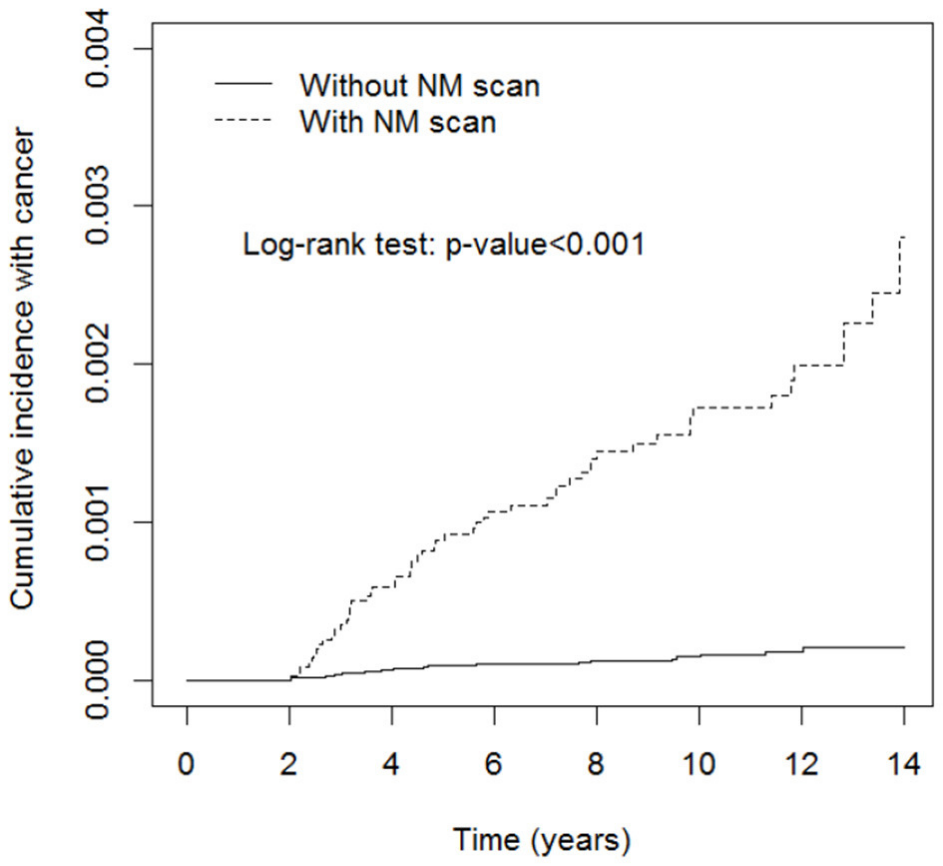
Cumulative incidence rate of pediatric neoplasms in patients with or without nuclear medicine (NM) scans. Pediatric neoplasms had a significantly higher incidence rate in the NM scan exposure group (log-rank test *p* < 0.001).

Table 3 shows the stratification of the number of NM exams in the exposed cohort into 1-2, 3-4, and 5 or more times and revealed that the incidence rates of pediatric neoplasms increased gradually with the increased frequency of NM exams (IR, 11.5; adjusted HR, 7.5; 95% CI, 4.29–13.1; IR, 25.8; adjusted HR, 15.9; 95% CI, 7.00–36.1; IR, 93.8; adjusted HR, 56.4; 95% CI, 28.8–110.3). Figure 2 shows that the cumulative incidence of pediatric neoplasms in children who underwent NM scanning was significantly higher than that in children who did not undergo scanning (log-rank test *p* < 0.001).

**Table 3:** Risk of pediatric neoplasm by NM scan frequency.

**Frequency of NM scan**	***N***	**Event**	**PY**	**IR**	**Crude HR (95% CI)**	**Adjusted HR (95% CI)**
None	141,152	20	1,300,566	1.54	1.00	1.00
1–2	30,055	32	278,243	11.5	7.47 (4.27–13.1)^***^	7.50 (4.29–13.1)^***^
3–4	3,606	8	30,987	25.8	16.8 (7.40–38.2)^***^	15.9 (7.00–36.1)^***^
≥5	1,631	15	15,998	93.8	59.9 (30.7–117.1)^***^	56.4 (28.8–110.3)^***^

*NM, nuclear medicine; PY, person-years; IR, incidence rate per 100,000 person-years; HR, hazard ratio; Adjusted HR, multivariable analysis including sex, age, urbanization*.

*** *p < 0.001*.

## Discussion

This current study shows that pediatric patients who are exposed to NM examinations have a significantly higher risk of developing neoplasms than non-exposed subjects. The incidence rates of any malignant tumor and benign brain and central nervous neoplasm in the NM scanning cohort were significantly higher than those of patients without scanning (16.9 vs. 1.54; adjusted HR, 10.9; 95% CI, 6.53–18.2). Subsequent analysis revealed the trend that with an increasing number of NM scans, the risk of pediatric neoplasms was aggregated (increase in adjusted HR from 7.50 to 56.4 with the increase in frequency from 1–2 to ≥5).

The most significant consequence of low-level radiation exposure in humans is cancer. The carcinogenic risk of radiation may be two to three times higher in children than in adults, although this risk varies with organ and tissue type (8). Cancer induction resulting from radiation exposure occurs in a stochastic manner: there is no threshold point, and the risk increases proportionally with the dose (17). The vulnerability of the pediatric population to ionizing radiation is due not only to the enhanced radiosensitivity of their tissues but also to a longer time period for the manifestation of stochastic radiation effects. First, our study results revealed that the incidence rate of pediatric neoplasms was significantly higher in the NM exposure group from the beginning of the follow-up period, which is consistent with the lack of a stochastic effect threshold. Furthermore, a higher risk of cancer is significantly associated with a higher frequency of NM exams, which indicates higher radiation dose exposure. This proportional probability of cancer occurrence is also compatible with the dose-dependent stochastic effect (Table 2; Figure 2). The abovementioned two results demonstrate the traditionally accepted linear and no-threshold model of the stochastic radiation effect.

Hereditary deformity attributed to low-dose radiation is the other example of a stochastic effect. Earlier studies linked prenatal medical radiation exposure to pediatric cancer in offspring, but there was no solid evidence for these conclusions (18, 19). Recent articles reviewed animal studies or applied different risk estimation models to discuss low-dose radiation exposure in nuclear medicine and the associated risk of cancer (20, 21). A PubMed-based literature search revealed a lack of large-scale investigations into childhood NM radiation exposure and subsequent cancer risk. One of the possible reasons is difficulties in pediatric NM procedure dose estimation. NM radiation dosimetry is challenging due to diversities in the uptake, retention, and clearance of variable radiopharmaceuticals. Furthermore, individual administered doses in children also differ depending on the patient's body mass, type of examination, available SPECT model, examination time, and patient cooperation (22, 23). Nevertheless, updated nuclear medicine drug decay half-lives are now available in ICRP Publication 107 (24). Based on these contemporary radiopharmaceutical data and the new biokinetic model, more accurate absorbed dose estimations are available for patients examined with NM procedures (25–27). With advances in absorbed dose estimation, we expect more quantitative knowledge about the risk of cancer associated with pediatric NM procedures in the future. At present, parents of pediatric patients are always anxious about the lifelong cancer risk associated with NM procedures. According to our study results, cancer risk associated with pediatric NM examinations should never be ignored. As a result of this issue, we advise physicians to take a more comprehensive approach and to consider the benefits of nuclear medicine as well as the potential risk associated with radiation exposure when deciding the best option for pediatric patients.

## Limitation

The HWDC datasets do not provide information about the clinical purpose of NM examinations. Thus, a screening effect and selection bias in the NM exposure cohort may have been present in this study. Pediatric patients who underwent NM procedures might receive additional radiation exposure, such as through routine plain films or other special examinations. We excluded high radiation dose procedures such as CT scan, radiotherapy, PET scan, and therapeutic NM procedures in both the exposure and unexposed groups, and this confounding factor might not be strong enough to affect the significance of our result. In addition, the NHIRD (National Health Insurance Research Database) cannot provide radiopharmaceuticals' dosage from each NM examination, the lack of the accurate data about radiopharmaceuticals decay (related to the accurate absorbed radiation and exposure) is our major study limitation.

Therefore, due to the lack of data about the actual absorbed radiation dose at each NM examination, our results indicating higher pediatric cancer risk associated with NM exposure should be interpreted with caution. Besides above mentioned limitation, prenatal information and family history of our participants were not included in the HWDC datasets, and hereditary or genetic effects of developing pediatric neoplasms could not be assessed.

In conclusion, this study benefits from a long follow-up period (2000–2018) and a large sample size (35,292 subjects with NM exposure) and shows that low-dose radiation exposure from NM examinations is significantly associated with a higher risk of cancer and benign central nervous tumors in pediatric patients. Nuclear medicine practitioners, including physicians and technologists, should show more initiative to protect patients from radiation and should be prudent in optimizing the radiation dose in pediatric NM procedures.

## Data Availability Statement

The dataset used in this study is held by the Taiwan Ministry of Health and Welfare (MOHW). The Ministry of Health and Welfare must approve our application to access this data. Any researcher interested in accessing this dataset can submit an application form to the Ministry of Health and Welfare requesting access. Please contact the staff of MOHW (Email: stcarolwu@mohw.gov.tw) for further assistance. All relevant data are within the paper. Requests to access the datasets should be directed to stcarolwu@mohw.gov.tw.

## Ethics Statement

The studies involving human participants were reviewed and approved by the Central Regional Research Ethics Committee, China Medical University, Taichung, Taiwan (CMUH109–109-REC2–031). Written informed consent from the participants' legal guardian/next of kin was not required to participate in this study in accordance with the national legislation and the institutional requirements.

## Author Contributions

M-KY and C-HK: conception and design. C-HK: administrative support. All authors contributed significantly, agreement with the content of the manuscript, collection and assembly of data, data analysis and interpretation, manuscript writing, and final approval of manuscript.

## Funding

This study was supported in part by Taiwan Ministry of Health and Welfare Clinical Trial Center (MOHW110-TDU-B-212-124004), China Medical University Hospital (DMR-109-231, DMR-110-089, DMR-111-105), Ministry of Science and Technology (MOST 110-2321-B-039-003). The funders had no role in the study design, data collection and analysis, the decision to publish, or preparation of the manuscript.

## Conflict of Interest

The authors declare that the research was conducted in the absence of any commercial or financial relationships that could be construed as a potential conflict of interest.

## Publisher's Note

All claims expressed in this article are solely those of the authors and do not necessarily represent those of their affiliated organizations, or those of the publisher, the editors and the reviewers. Any product that may be evaluated in this article, or claim that may be made by its manufacturer, is not guaranteed or endorsed by the publisher.
